# Case report: successful endoscopic treatment of a large bowel perforation caused by chicken bone ingestion

**DOI:** 10.1097/MD.0000000000018111

**Published:** 2019-12-16

**Authors:** Miroslav Simunic, Ivan Zaja, Zarko Ardalic, Radoslav Stipic, Marina Maras-Simunic

**Affiliations:** a Department of Gastroenterology; b Department of Surgery; c Department of Radiology, University Hospital Centre Split, Split, Croatia.

**Keywords:** colonoscopy, foreign body, sharp objects

## Abstract

**Rationale:**

Large bowel perforations by a foreign body are rarely diagnosed pre-operatively due to non-specific clinical symptoms. The safety and efficacy of foreign body removal via upper endoscopy is well-established and strongly recommended. There is far less experience of endoscopic treatment of sharp foreign bodies impacted in lower parts of gastrointestinal tract.

**Patient concerns:**

The patient was 78-year-old female with abdominal pain and nausea. Symptoms had begun 48 hours prior to hospital admission. She had lost over 10 kg of body weight in the previous couple of months

**Diagnosis:**

A multidetector-row computed tomography (MDCT) examination of the abdomen revealed mural thickening and enhancement of the cecum with haziness and linear areas of high attenuation in the pericecal fat tissue. A colonoscopy showed, the clear presence of a sharp 5.5-cm-long chicken bone perforating the cecal wall at the antemesenteric site close to the Bauchini valve.

**Interventions:**

A quarter of the bone that had penetrated the cecal wall was pulled out with a flexible colonoscopy using a polypectomy snare. Due to the form and length of the bone, it was withdrawn through the entire colon, using pointed end trailing.

**Outcomes:**

The patient was discharged three days after colonoscopy with normal laboratory results and without any pain.

**Lessons:**

In cases where sharp foreign bodies stuck into the large bowel, it is highly advisable to try to remove them via colonoscopy, before deciding to resolve the issue through a surgical intervention.

## 1 Introduction

Foreign body ingestion is frequent in everyday clinical practice and mostly occurs in the pediatric population. In most cases (80%–90%), these swallowed foreign bodies pass through the gastrointestinal (GI) tract without causing any harm. There is, however, significant morbidity associated with retained foreign bodies as they may cause GI mucosal erosion, ulceration, bleeding, local scarring or perforation, depending on the size, shape, and location of the foreign body within the GI tract.^[1]^ In adults, most foreign body ingestions are related to food consumption that leads to either bone or meat bolus impaction. Approximately 10% to 20% of patients who ingest foreign bodies will require endoscopic treatment and 1% of all patients require urgent surgical treatment.^[2]^ The safety and efficacy of foreign body removal via upper endoscopy are well-established, emergent endoscopy for sharp foreign bodies in the esophagus and urgent endoscopy for those discovered in the stomach or duodenum are thus suggested.^[3]^ Endoscopic treatment for sharp foreign bodies that are out of reach of upper endoscopy are less common, and there is no generally accepted recommendation on how to treat these patients. Here we present the case of a 78-year-old patient with a large bowel perforation caused by chicken bone ingestion; the patient was successfully managed via a colonoscopic approach.

## 2 Case presentation

A 78-year-old female presented to the surgical emergency unit at the University Hospital with abdominal pain and nausea. Symptoms had begun 48 hours prior to hospital admission. She had lost over 10 kg of body weight in the previous couple of months, hence a diagnosis of gastrointestinal malignancy was suspected. Her vital signs were: RR, 140/90 mmHg; pulse rate, 90 beats/min; respiration rate, 15/min; and body temperature, 38.1°C. Her past medical history revealed only arterial hypertension. She did not mention having ingested the foreign body.

Physical examination pointed to a distended and diffusely tender abdomen with right lower abdomen rebound. Laboratory tests were as follows: white blood cell count, 11.6 × 10^9^/L; serum creatinine level, 58 μmol/L; blood urea nitrogen level, 2.5 mmol/L; blood glucose level, 6.1 mmol/L; sodium 137 mmol/L; potassium 4.4 mmol/L; CRP 76.6 mg/L. An urgent abdominal X-ray did not show any sign of ileus or perforation.

**Figure 1:**
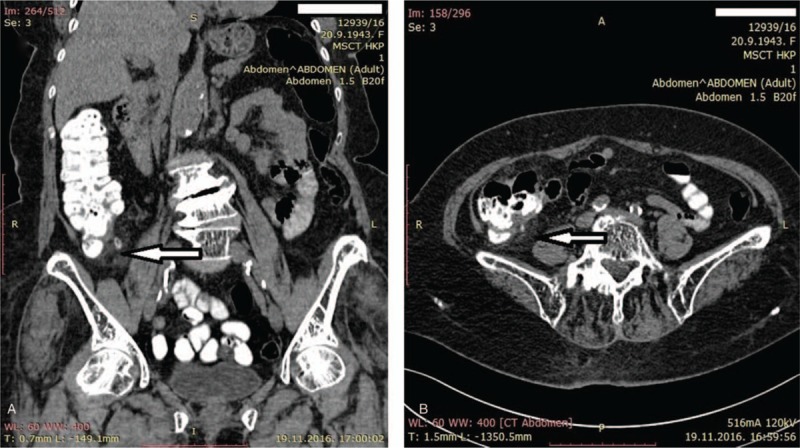
MSCT scans of the abdomen. Thickening of the cecal wall and increase in of the pericolic fatty tissue density on a 5x3 cm area, corresponding to the diffuse inflammatory infiltration. There are no signs of abscess. Appendix is normal in morphology. A. transverse MSCT plane; B. coronal MSCT plane.

A multidetector-row computed tomography (MDCT) examination of the abdomen was performed using a 64-detector-row CT scanner (Siemens Somatom Definition AS, Erlangen, Germany). Scans were performed before and after the administration of intravenous contrast, with prior peroral diluted iodine contrast uptake, and images were analyzed in axial and reconstructed coronal and sagittal planes. MDCT revealed mural thickening and enhancement of the cecum with haziness and linear areas of high attenuation in the pericecal fat tissue (Fig. 1). The terminal ileum and ileocecal valve had a normal appearance. The wall thickness and outer diameter of the appendix were normal, having neither air nor appendicolith in the lumen. There was no periappendiceal abscess, phlegmon, extra-luminal air, or enlarged lymph nodes. Additionally, a foreign body in the lumen of the colon was not seen. Due to MDCT signs of mural thickening and enhancement of the cecum with haziness and linear areas of high attenuation in the pericecal fat tissue as well as due to increased inflammatory laboratory parameters, the patient was hospitalized and treated with broad-spectrum antibiotics (ciprofloxacin and metronidazole) and crystalloid fluids.

**Figure 2:**
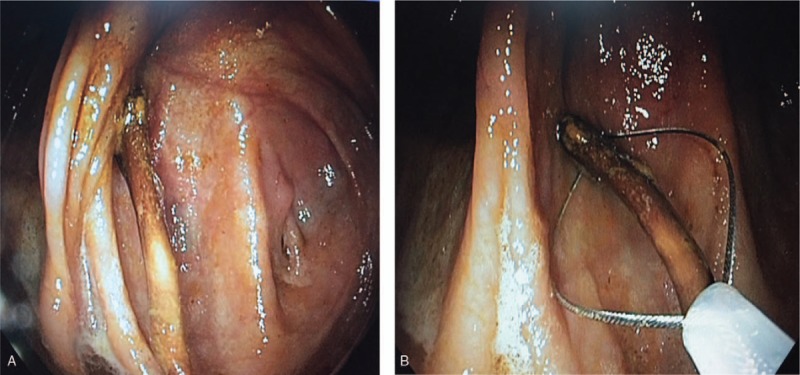
Colonoscopy findings. A. Caudal to Bauchini valve, clear presence of a sharp chicken bone that perforated the cecal wall was seen. B. Bone extraction was performed with a polypectomy snare.

**Figure 3:**
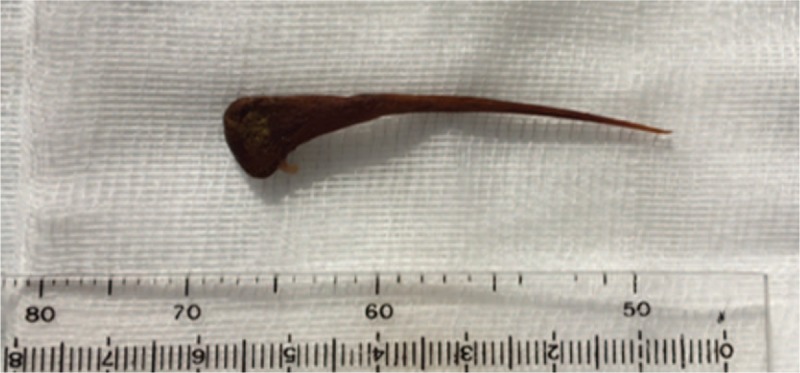
Extracted sharp chicken bone measures 5.5 cm in length.

The day after admission, colonoscopy under conscious sedation was performed following oral bowel preparation. Close to the Bauchini valve, the clear presence of a sharp 5.5-cm-long chicken bone perforating the cecal wall at the antemesenteric site was seen. The mucosa of the remainder of the cecum had a normal appearance (Fig. 2A). A quarter of the bone that had penetrated the cecal wall was pulled out using a polypectomy snare (Fig. 2B). Due to the form and length of the bone, we continued withdrawing it through the entire colon, using pointed end trailing. During the entire removal process, we insufflated air in the bowel (maintaining a wide lumen) and positioned the bone to the center of the visual field to avoid mucosal injury. The slight curvature of the bone was helpful for passing the splenic and hepatic flexures without any damage (Fig. 3). Post-procedure treatment with fluid resuscitation and broad-spectrum antibiotics (ciprofloxacin and metronidazole) was successful, and the patient was discharged three days after colonoscopy with normal laboratory results and without any pain.

## 3 Discussion

The most common gastrointestinal foreign objects are food boluses. Children and older people are at an especially high risk of foreign body ingestion. Studies performed before the era of flexible endoscopy revealed that the majority (80%–90%) of foreign bodies spontaneously pass through the GI tract without causing any significant damage, suggesting conservative treatment as the preferred method of management. Therefore, endoscopic methods are not indicated as the first choice, the patients are mostly observed anticipating spontaneous passage of the foreign body.^[4,5]^ However, considerable advances in endoscopic methods and devices have resulted in lowering the threshold for diagnostic endoscopy and shortening the time span between ingestion and initiating endoscopy with a stand-by setup that allows the procedure to be converted into an intervention.^[6]^ If not promptly and adequately treated, these emergencies can result in significant morbidity. Nevertheless, when intentional ingestion happens, there are much more frequent both endoscopic (63%–76%), and surgical (12%–16%) interventions. Fortunately, mortality rates have been extremely low (<0.1%).^[2]^ Impactions mostly occur at the site of physiological narrowing of the upper GI tract (upper esophagus, cardia, pylorus, etc.). Objects larger than 2 cm in length infrequently pass through the pylorus or ileocecal valve, and those larger than 5 cm in length rarely pass by the duodenal sweep. Pre-existing GI abnormalities increase the risk of complications (e.g., stenosing lesions, diverticula, fistulas.).^[7,8]^ The majority of studies evaluating treatment strategies and their outcomes are retrospective. Therefore, patients are still treated on a case-by-case basis when making decisions regarding diagnostic radiological exams, the endoscopic methods to retrieve the foreign body, and when determining the surveillance approach, depending of the type, size, and shape of the foreign body as well as the symptoms and signs of complications.^[9]^

We presented a remarkably rare case of cecal perforation by a sharp and unusually long chicken bone, which was not visible in imaging studies. In the plain abdominal X-ray image, the thin bone was covered with other intestinal contents which it could not be distinguished from, since the density in such an X-ray image is the result of the sum of all densities – feces, gas and foreign body.

In abdominopelvic MDCT, a hyperdense iodine oral contrast covered the intestinal contents, and the foreign body as well. Our MDCT acquisition protocol was tailored to the working diagnosis of suspected bowel pathology such as neoplasm or inflammation. In such cases, positive oral agents hold an advantage when compared to either the use of neutral oral contrast or non-use of oral contrast prior to scanning, primarily in the diagnostics of thickening of the bowel wall, pericolic inflammation or fluid collections.^[10]^

Had there been a clinical suspicion of a foreign body in the digestive tract, it might have been more appropriate to apply the CT protocol without administration of the iodine oral contrast, which can simplify the detection of a radiopaque foreign body.^[11]^

Most cases including perforation require urgent surgery; however, in this case, the endoscopic treatment was curative. Although only 1% of swollen foreign bodies cause perforation, in literature there are many reports of foreign bodies that caused open perforation of large or small bowel with acute symptoms of peritonitis.^[10]^ Perforations with an acute clinical picture are much more frequent than perforations with localized immune response and mild symptoms. Patients with acute symptoms are, as a rule, urgently operated, regardless of whether the operations were indicated by evidence of foreign body, or in other cases, when the foreign body is found intraoperatively.^[8,11,12]^

In our opinion, the patient swallowed a chicken bone a few days before the first symptoms. The symptoms appeared right after the foreign body got stuck in the wall of the cecum and aggravated by local inflammation at the penetration site. There was no open perforation so the symptoms amplified gradually. We assume that colonoscopic intervention for our patient would have been the first choice as well, in case the existence of the foreign body had been verified before the intervention, considering that peritonitis, pneumoperitoneum or obstruction of the intestine were not confirmed at the same time.

Hershman et al^[9]^ described 2 cases of sharp foreign body removal from the large bowel via colonoscopy, suggesting that it is highly advisable to perform an endoscopic method before deciding to resolve the issue through a surgical intervention. In their literature search, they found only three reports with no need for any surgical assistance: the successful removal of a 3-cm dental needle in the cecum,^[13]^ a toothpick impacted in the rectosigmoid wall,^[14]^ and the mucosal bridge entrapping a plastic twist-tie.^[15]^ The devices used did not differ from those used for endoscopic management for upper GI tract foreign body management, and they included polypectomy snares and retrieval forceps or baskets. We decided to pull the chicken bone out with the sharp end trailing because of its shape and length, while permanently insufflating air to keep the bowel lumen wide open and placing the bone in the center of it. Hershman et al suggested a different strategy of grasping and covering the sharp end of the foreign body and withdrawal with the blunt end trailing, similar to removal of foreign bodies in the upper GI tract.

Although this patient had some clear benefits from endoscopic removal, further experience is needed to make a uniform decision when deciding the best approach for each case by combining clinical, laboratory and radiological findings and choosing the best endoscopic techniques.

## 4 Conclusion

Foreign bodies in the GI tract mostly pass without pathological consequences. Large bowel perforations by foreign bodies are rarely diagnosed preoperatively due to non-specific clinical symptoms that are similar to frequent surgical conditions, such as diverticulitis and appendicitis. The risk of perforation depends on the length and sharpness of the object. Most perforations occur at the site of narrowing and angulation of the GI tract. Intestinal perforation by a chicken bone is rare, especially in lower parts of the GI tract. Treatment usually involves surgical intervention and bowel resection; however, in the present case, endoscopic removal was the definitive treatment.

## Author contributions

**Conceptualization:** Miroslav Simunic.

**Data curation:** Miroslav Simunic, Radoslav Stipic, Marina Maras-Simunic

**Investigation:** Ivan Zaja, Zarko Ardalic.

**Supervision:** Marina Maras-Simunic

**Writing – original draft:** Miroslav Simunic, Ivan Zaja.

**Writing – review & editing:** Miroslav Simunic.
